# Tenofovir alafenamide for prevention of HBV reactivation in HBsAg-negative, anti–HBc-positive patients undergoing rituximab-based chemotherapy: A multicenter randomized controlled trial

**DOI:** 10.1097/HC9.0000000000000859

**Published:** 2025-12-03

**Authors:** Sanam Tabataba Vakili, Grishma Hirode, Atif Zahoor, Ahreni Saunthar, Joshua B. Feld, Bettina E. Hansen, Ambreen Syeda, Harry L.A. Janssen, Vishal Kukreti, John Kuruvilla, Anca Prica, Matthew Cheung, Rena Buckstein, Lisa Hicks, Carla S. Coffin, Lesley E. Street, Patricia Disperati, Kelvin K. Chan, Michael Crump, Jordan J. Feld

**Affiliations:** 1 Toronto Centre for Liver Disease, University Health Network, Toronto, Ontario, Canada; 2 Viral Hepatitis Care Network (VIRCAN), University Health Network, Toronto, Ontario, Canada; 3 Erasmus University Medical Centre, Rotterdam, The Netherlands; 4 Princess Margaret Cancer Centre, Toronto, Ontario, Canada; 5 Sunnybrook Odette Cancer Centre, University of Toronto, Toronto, Ontario, Canada; 6 Division of Hematology and Oncology, St. Michael’s Hospital, Toronto, Ontario, Canada; 7 Division of Gastroenterology and Hepatology, Department of Medicine, Cumming School of Medicine, University of Calgary, Calgary, Alberta, Canada; 8 Division of Hematology and Hematologic Malignancies, Cumming School of Medicine, University of Calgary, Calgary, Alberta, Canada; 9 Michael Garron Hospital, Toronto, Ontario, Canada

**Keywords:** anti-HBc positive, chemotherapy, HBsAg negative, HBV, hepatitis B, lymphoma, reactivation, rituximab, tenofovir alafenamide

## Abstract

**Background and Aims:**

Immunosuppression can cause hepatitis B virus (HBV) reactivation, leading to severe outcomes in patients with “resolved” HBV infection. This multicenter, randomized, placebo-controlled trial assessed the efficacy of preemptive antiviral therapy in HBsAg-negative, anti–HBc-positive patients receiving rituximab-based chemotherapy for non-Hodgkin lymphoma (NHL).

**Methods:**

Patients were randomized 1:1 to tenofovir alafenamide (TAF)/placebo across 3 phases: chemotherapy plus TAF/placebo (phase 1), TAF/placebo post-chemotherapy (phase 2), and follow-up after therapy cessation (phase 3). The primary endpoint was HBsAg reverse seroconversion. HBsAg and ALT were monitored every 3–12 weeks, depending on treatment phase, and HBV DNA was measured post hoc. ClinicalTrials.gov (NCT02186574).

**Results:**

Among 42 patients (median age 65.2 years, 52.4% male, 52.4% aggressive lymphoma, 73.8% anti-HBs positive), 20 received TAF and 22 received a placebo. Median ALT was 20.0 U/L (IQR: 15.0–28.0) at baseline. Median follow-up was 69.4 weeks (IQR: 63.7–166), with 6.1 weeks (IQR: 4.7–8.3) between visits. During follow-up, 2 patients in the TAF arm, but none receiving placebo, experienced HBsAg reverse seroconversions: occurring in phase 3 at 62.3 weeks from baseline, and in phase 1 at 20.0 weeks from baseline. Neither patient experienced ALT >2× ULN. HBV DNA >1000 IU/mL was observed in 8 instances among 6 patients, 3 in each arm, with no associated hepatitis. Low-level DNA (<1000 IU/mL) was not indicative of reverse seroconversion, DNA increases, or ALT elevations.

**Conclusions:**

The use of preemptive TAF therapy did not reduce the risk of HBsAg reverse seroconversion; however, the findings should be interpreted with caution as the study was underpowered due to slow enrolment leading to early termination. Low-level HBV DNA elevations were not associated with HBV reactivation. Thus, close HBsAg and ALT monitoring are adequate in HBsAg-negative patients undergoing rituximab-based chemotherapy.

## Graphical abstract

**Figure:**
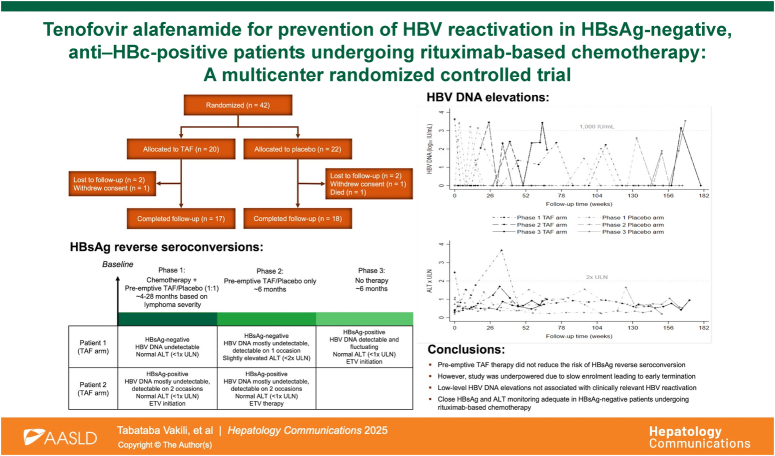

## INTRODUCTION

Hepatitis B virus (HBV) remains a public health problem with over 250 million people chronically infected and up to 2 billion people with prior exposure worldwide.^1^,^2^ Hepatitis B surface antigen (HBsAg) clearance is associated with a very good long-term prognosis and minimal risk of liver-related complications.^3^,^4^,^5^,^6^ However, HBV can persist in the form of covalently closed circular DNA within the nuclei of hepatocytes.^7^,^8^ The disease remains clinically silent because of successful immune control of viral replication with antibodies to HBV core antigen (anti-HBc), with or without antibodies to hepatitis B surface antigen (anti-HBs), as the only markers of exposure.^9^ Significant immunosuppression may disrupt immune control, leading to increased HBV replication and HBsAg reappearance.^10^

While HBsAg-negative, anti–HBc-positive patients have a low risk of HBV reactivation with standard chemotherapy, adding rituximab, a monoclonal antibody against CD20, or other CD20-depleting agents, increases the risk of HBsAg reappearance, known as reverse seroconversion.^11^,^12^,^13^ Shortly after the introduction of rituximab, cases of HBsAg reverse seroconversion with HBV-associated hepatitis were reported, many of which were severe or even fatal.^11^,^12^ Rituximab is used to treat indolent and aggressive non-Hodgkin lymphoma (NHL), and in several chronic immunologically mediated diseases such as refractory rheumatoid arthritis, vasculitis, and other conditions.^14^,^15^,^16^ Screening for HBV infection with HBsAg, anti-HBs, and anti-HBc is recommended in patients scheduled to start immunosuppressive therapy.^17^,^18^,^19^,^20^

Compared with on-demand therapy, preemptive antiviral therapy using agents like lamivudine, entecavir (ETV), and tenofovir has proven effective for HBsAg-positive patients undergoing chemotherapy or immunosuppressive therapy.^18^,^19^ Thus, guidelines recommend antiviral therapy to prevent HBV reactivation in HBsAg-negative, anti–HBc-positive patients receiving anti–CD20-based therapy. However, supporting data are limited, and the optimal monitoring strategy remains unknown. While studies show that preemptive therapy prevents HBV DNA reappearance, the significance of low-level elevations (<1000 IU/mL) in the absence of HBsAg or evidence of hepatitis is unclear, and the clinical benefit of preemptive therapy, especially using tenofovir alafenamide (TAF), in these patients remains uncertain.^21^ TAF is a newer nucleos(t)ide analogue with improved renal and bone safety profiles compared with tenofovir disoproxil fumarate (TDF).^22^,^23^

This study aimed to assess the efficacy of preemptive TAF therapy in preventing clinically significant HBV reactivation in patients with “resolved” HBV infection (HBsAg-negative, anti–HBc-positive) receiving rituximab-based chemotherapy for lymphoma. A secondary aim was to evaluate the utility of serial HBV DNA testing versus HBsAg and ALT monitoring alone.

## METHODS

### Study design and participants

In this multicenter, double-blind, randomized, placebo-controlled trial, adult patients were recruited from 5 hospitals in Toronto and Calgary, Canada, between May 2015 and April 2020. The Toronto Centre for Liver Disease was the coordinating center. Eligible participants were HBsAg-negative and anti–HBc-positive with indolent or aggressive NHL scheduled for anti–CD20-based chemotherapy (rituximab or obinutuzumab).

Main exclusion criteria included therapy with known activity against HBV, baseline ALT >10× upper limit of normal (ULN), or ALT between 2 and 10× ULN with HBV DNA >2000 IU/mL, and life expectancy <3 months, coinfection (human immunodeficiency virus, hepatitis C virus), creatinine clearance <15 mL/min, and intolerance to TAF. This study was approved by the research ethics boards of all participating centers and registered with ClinicalTrials.gov (NCT02186574). All study procedures were conducted in accordance with the ethical principles outlined in the Declarations of Helsinki and Istanbul. All patients provided written informed consent before enrollment.

### Randomization and blinding

Participants were randomized (1:1) to receive TAF or placebo using a computer-generated randomization schedule in blocks of 4 and stratified by anti-HBs status and lymphoma type (indolent vs. aggressive). Both investigators and participants remained blinded to treatment assignment.

### Endpoints

The primary endpoint was the difference in the proportion of HBsAg reverse seroconversion between study arms. Secondary endpoints included HBV reactivation, HBV-associated hepatitis, HBV-associated liver failure, TAF safety, lymphoma-related mortality, and all-cause mortality.

### Definitions

HBsAg reverse seroconversion was defined as HBsAg detection during follow-up with a lower limit of detection (LLOD) of 0.05 IU/mL. HBV reactivation was defined as reverse seroconversion or HBV DNA reappearance in a patient with undetectable baseline HBV DNA, or a >1 log_10_ IU/mL increase in those with detectable baseline DNA. HBV-associated hepatitis was defined as ALT >2× ULN with HBV reactivation. HBV-associated liver failure was defined as HBV reactivation with HBV DNA ≥1000 IU/mL and bilirubin >2× ULN, INR >2.0, or clinical evidence of new-onset portal hypertension (ascites) or hepatic encephalopathy. ULN for ALT was 40 U/L.

### Monitoring and measurements

The study follow-up occurred in 3 phases. In phase 1, patients received chemotherapy and TAF or placebo for the first 4–28 months. The duration of therapy was determined by underlying lymphoma type: 6×3-week cycles for aggressive lymphoma, and 6×3–4 week cycles followed by 2 years of maintenance therapy every 3 months for indolent lymphoma. In phase 2, after completing chemotherapy, patients received TAF or a placebo for 6 months. In phase 3, study therapy was withdrawn, and patients were monitored for another 6 months. Open-label ETV without unblinding was started in patients with HBV reactivation who experienced HBsAg reverse seroconversion. Patients were monitored every 3–4 weeks during chemotherapy, and every 6 weeks thereafter. Adherence was assessed at each visit by direct inquiry regarding missed doses, side effects, and medication-related challenges; no objective measures such as pill counts or pharmacy refill data were collected.

Laboratory measurements included HBsAg, anti-HBc, and anti-HBs using the Abbott Architect, complete blood count, liver panel (ALT, AST, bilirubin, albumin, INR), creatinine, pregnancy testing, and urinalysis. A research sample was stored at −80 °C at each visit. HBsAg was measured every 3–12 weeks, depending on treatment phase, until 12 months after chemotherapy. HBV DNA was not measured in real-time unless HBsAg reverse seroconversion occurred. Per protocol, real-time HBV DNA testing was permitted in cases of HBsAg reverse seroconversion and/or significant ALT elevation (≥120 U/L), total bilirubin >40 µmol/L, or INR >1.3. In such cases, patients underwent clinical evaluation and repeat liver enzyme testing (ALT, AST, ALP), as well as liver function tests (bilirubin, albumin, INR), along with HBsAg and HBV DNA testing. HBV DNA was measured post-hoc from stored samples using the Abbott Alinity assay with an LLOD of 20 IU/mL.

### Statistical analysis

Based on prior literature and conservative estimates of 0.5% and 15% for HBsAg reverse seroconversion in the TAF and placebo arms, respectively, 59 patients per arm were required to achieve 80% power to detect a difference between study arms with α=0.05, accounting for 5% dropout per arm. The study was prematurely stopped due to slow enrollment.

Patient characteristics were summarized as means with standard deviation (SD) or medians with interquartile range (IQR) for continuous variables, and proportions for categorical variables. Patients were grouped by lymphoma type as indolent lymphomas, including chronic lymphocytic leukemia (CLL), and aggressive lymphomas, due to differences in treatment regimens. The main outcome was analyzed using the intention-to-treat (ITT) principle. Statistical analyses were performed using STATA Version 15.1 (StataCorp, College Station, TX).

## RESULTS

**FIGURE 1:**
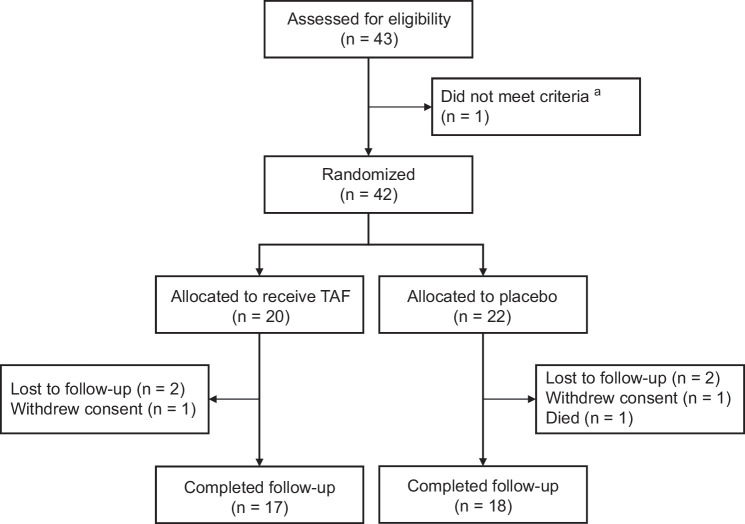
CONSORT diagram. ^a^ This patient did not meet eligibility due to a creatinine clearance of <15 mL/min. Abbreviation: TAF, tenofovir alafenamide.

Prior to study discontinuation, 42 patients were enrolled (20 received TAF, 22 placebo) (Figure 1). Median age was 65.2 years (IQR: 58.4–70.2), 22 (52.4%) were male, 18 (42.9%) were White, 19 (45.2%) were Asian, 22 (52.4%) had aggressive lymphomas, and 29 (69.1%) were anti-HBs positive at baseline (Table 1). At baseline, median ALT was 20.0 U/L (IQR: 15.0–28.0), total bilirubin 6.5 µmol/L (IQR: 4.5–11.0), and creatinine 72.0 µmol/L (IQR: 61.0–84.0). Median follow-up was 69.4 weeks (IQR: 63.7–166) with 6.1 weeks (IQR: 4.7–8.3) between visits. TAF was well-tolerated with no significant adverse events or drug-related toxicity. In addition, renal function did not differ between treatment groups (Supplemental Figure S1, http://links.lww.com/HC9/C199).

**TABLE 1:** Patient characteristics by arm

	Total (N=42)	TAF (n=20)	Placebo (n=22)
At baseline			
Age, y, median (IQR)	65.2 (58.4–70.2)	66.6 (60.5–70.1)	62.7 (57.5–70.2)
Male, n (%)	22 (52.4)	8 (40.0)	14 (63.6)
Race, n (%)			
White	18 (42.9)	7 (35.0)	11 (50.0)
Black	2 (4.8)	2 (10.0)	0 (0)
Asian	19 (45.2)	10 (50.0)	9 (40.9)
Other	3 (7.1)	1 (5.0)	2 (9.1)
Lymphoma type, n (%)			
Indolent	20 (47.6)	11 (55.0)	9 (40.9)
Aggressive	22 (52.4)	9 (45.0)	13 (59.1)
ALT, U/L, median (IQR)	20.0 (15.0–28.0)	19.0 (15.0–25.0)	22.5 (14.0–29.0)
ALT, ULN, median (IQR)	0.5 (0.4–0.7)	0.5 (0.4–0.6)	0.6 (0.4–0.7)
Direct bilirubin ^a^, µmol/L, median (IQR)	2.5 (2.0–5.0)	2.0 (2.0–3.0)	4.0 (2.0–6.0)
Total bilirubin ^a^, µmol/L, median (IQR)	6.5 (4.5–11.0)	6.5 (4.0–7.0)	7.0 (5.0–13.0)
Creatinine, µmol/L, median (IQR)	72.0 (61.0–84.0)	71.0 (57.0–80.0)	72.0 (64.0–85.0)
Anti-HBs positive, n (%)	31 (73.8)	14 (70.0)	17 (77.3)
HBV DNA detectable, n (%)	1 (2.4) ^b^	1 (5.0) ^b^	0 (0)
During follow-up			
Total time, weeks, median (IQR)	69.4 (63.7–166)	69.4 (60.4–169)	69.6 (65.0–166)
Time between visits, weeks, median (IQR)	6.1 (4.7–8.3)	6.5 (5.0–8.5)	5.6 (4.6–7.7)
Number of visits, median (IQR)	15 (12–20)	15 (12–20)	15 (13–21)

^a^ Direct bilirubin was missing for 12 patients, and total bilirubin was missing for 2 patients.

^b^ Quantitative HBV DNA at baseline was 4260 IU/mL for this patient.

Abbreviations: ALT, alanine aminotransferase; Anti-HBs, anti-hepatitis B surface antibodies; TAF, tenofovir alafenamide; ULN, upper limit of normal.

### HBsAg reverse seroconversion

**FIGURE 2:**
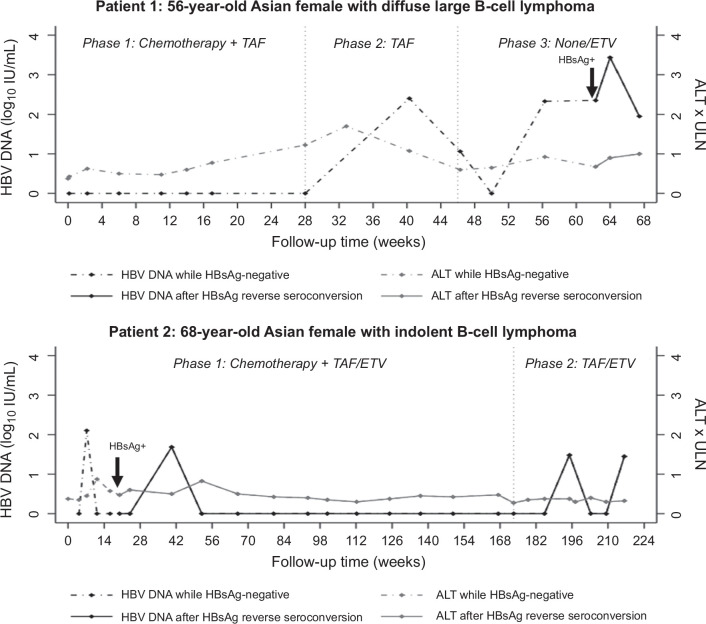
Patients with HBsAg reverse seroconversions. Abbreviations: ALT, alanine aminotransferase; ETV, entecavir; HBsAg, hepatitis B surface antigen; HBV, hepatitis B virus; NHL, non-Hodgkin lymphoma; TAF, tenofovir alafenamide; ULN, upper limit of normal.

During follow-up, 2 patients in the TAF arm, but none receiving placebo, experienced HBsAg reverse seroconversions (Figure 2).

Patient 1 was a 56-year-old Asian female with diffuse large B-cell lymphoma. HBV DNA became detectable (251 IU/mL) for the first time during phase 2 at 40.3 weeks from baseline (23.3 weeks after stopping chemotherapy). Peak ALT (68 U/L) also occurred in phase 2 at 32.9 weeks from baseline. HBV DNA remained detectable at the next visit (46.3 weeks from baseline), which was the first visit in phase 3 after TAF discontinuation and fluctuated between 212 and 224 IU/mL thereafter, until HBsAg became positive at 62.3 weeks from baseline. Peak HBV DNA level was 2720 IU/mL ~2 weeks after HBsAg reverse seroconversion, at which point ETV was started, and HBV DNA was suppressed to undetectable levels.

Patient 2 was a 68-year-old Asian female with indolent B-cell lymphoma. ALT remained normal (peak: 35 U/L) during phase 1. HBsAg was detected during phase 1 at 20.0 weeks from baseline, at which point ETV was started. HBV DNA was detectable on 4 occasions: during phase 1 at 7.3 weeks (127 IU/mL) and at 40.3 weeks (48.5 IU/mL) from baseline, and during phase 2 at 195 weeks (30.4 IU/mL) and 216 weeks (28.1 IU/mL) from baseline. The latter 3 instances occurred after ETV initiation (during which time the patient should have been taking both ETV and TAF).

Neither patient with reverse seroconversion experienced ALT >2x ULN or other sequelae or experienced any symptoms or showed signs of acute or chronic liver injury. HBsAg remained positive with suppressed HBV DNA on open-label therapy at the end of the study.

### HBV DNA elevations

**FIGURE 3:**
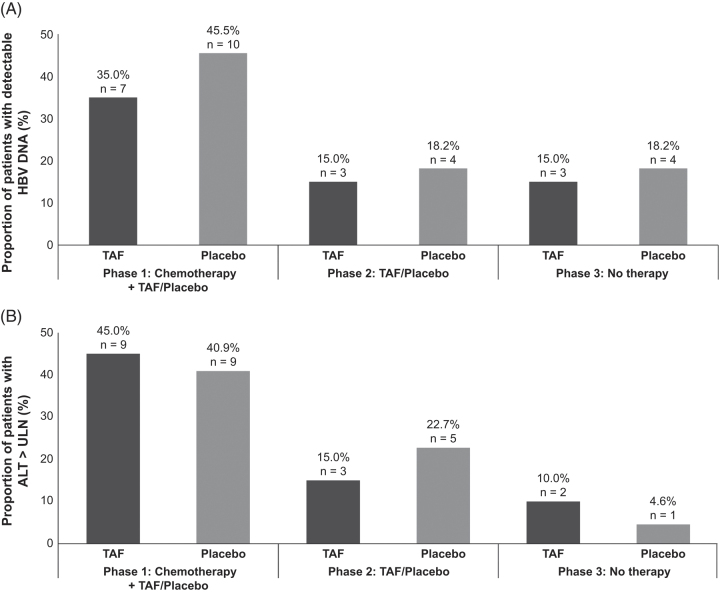
Patients with detectable HBV DNA and elevated ALT levels by arm. Abbreviations: ALT, alanine aminotransferase; HBV, hepatitis B virus; TAF, tenofovir alafenamide; ULN, upper limit of normal.

At baseline, HBV DNA was detectable in one TAF-arm patient and none of the placebo-arm patients (Table 1). During the study, 31 episodes of detectable HBV DNA occurred in 10 TAF-arm patients (peak: 4260 IU/mL) and 29 episodes in 11 placebo-arm patients (peak: 3440 IU/mL) (Figure 3A and Supplemental Figure S2, http://links.lww.com/HC9/C199). ALT levels remained largely within normal limits during follow-up for both arms (Figure 3B and Supplemental Figure S3, http://links.lww.com/HC9/C199). HBV DNA detectability was not associated with future HBsAg reappearance or ALT elevation.

**FIGURE 4:**
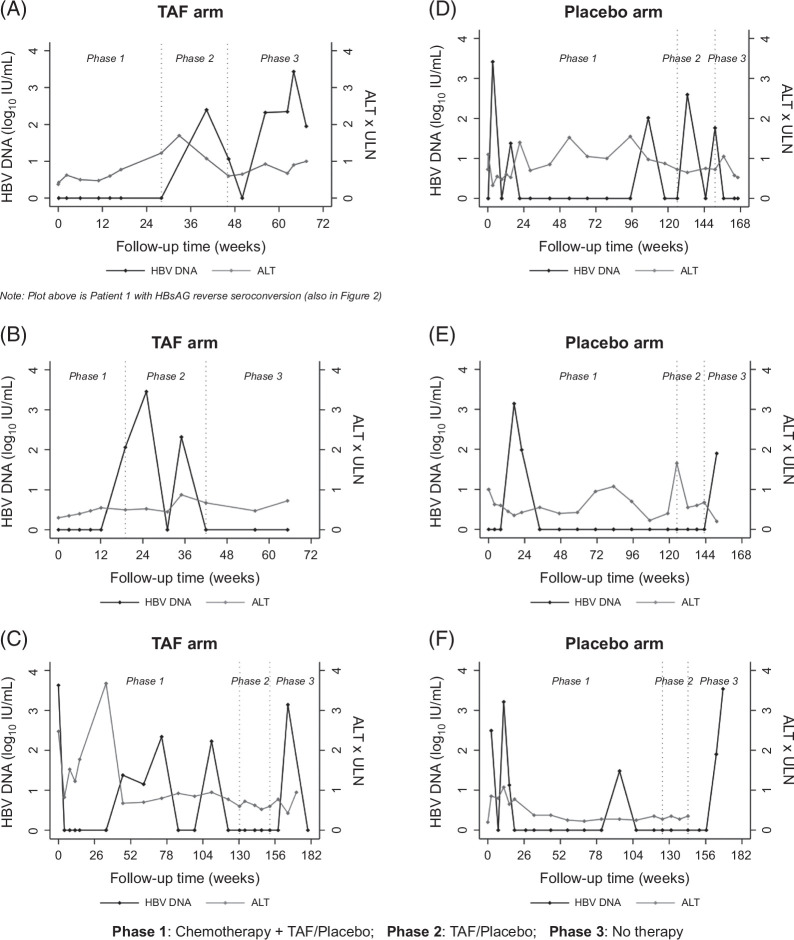
Quantitative HBV DNA and ALT levels among patients with HBV DNA >1000 IU/mL. Abbreviations: ALT, alanine aminotransferase; HBV, hepatitis B virus; TAF, tenofovir alafenamide; ULN, upper limit of normal.

HBV DNA >1000 IU/mL was observed in 8 instances among 6 patients, 3 in each arm (Figure 4). Among these 8 instances, 1 occurred at baseline (Figure 4C), 3 during phase 1 in the placebo arm (Figures 4D–F), 1 during phase 2 in the TAF arm (Figure 4B), 1 during phase 3 in the placebo arm (Figure 4F), and 2 during phase 3 in the TAF arm (Figures 4A, C) of which 1 occurred after HBsAg reverse seroconversion. Two patients experienced HBV DNA >1000 IU/mL on 2 separate occasions (Figures 4C, F). Peak HBV DNA (4260 IU/mL) in the TAF arm occurred at baseline alongside elevated ALT (99 U/L) (Figure 4C). This same individual had elevated HBV DNA (1390 U/L) again during phase 3 (after TAF cessation) at 165 weeks from baseline with normal ALT (17 U/L). The second patient had elevated HBV DNA (1630 IU/mL) during phase 1 at 11.4 weeks from baseline, and then again during phase 3 at 168 weeks (3440 IU/mL) from baseline with no accompanying ALT elevations (Figure 4F).

No other patients had an ALT >2x ULN despite HBV DNA >1000 IU/mL. Anti-HBs was positive for 6 of the 8 instances of HBV DNA >1000 IU/mL, negative at 1 instance, and unknown for 1 instance. There was no HBV DNA threshold that resulted in future HBsAg reverse seroconversion or ALT elevation. Patients with ≥1 episode of HBV DNA >1000 IU/mL were younger and more often female compared with the total cohort with normal median ALT at the time of HBV DNA elevation (Supplemental Table S1, http://links.lww.com/HC9/C199).

### ALT elevations

At baseline, 2 TAF-arm patients and 3 placebo-arm patients had ALT > ULN. During the study, 38 episodes of ALT > ULN occurred in 10 TAF-arm patients (peak: 396 U/L), and 28 episodes in 10 placebo-arm patients (peak: 123 U/L) (Figure 3B and Supplemental Figure S3, http://links.lww.com/HC9/C199). One TAF-arm individual with elevated ALT (99 U/L) at baseline had simultaneously high HBV DNA (4260 IU/mL) (described previously). HBV DNA was detectable in 6 other instances of ALT > ULN during follow-up. In 5 of the 6 instances, ALT levels were between 43 and 116 U/L, and HBV DNA levels remained <1000 IU/mL (range 13.9–872 IU/mL). In the one instance with HBV DNA >1000 IU/mL, ALT was 43 U/L (described previously).

### Secondary outcomes

No episodes meeting the definition of HBV-associated hepatitis or liver failure were documented. No serious adverse events were related to the intervention, and no deaths were reported in the TAF arm. One non–liver-related death occurred in the placebo arm: a cardiac arrest during phase 1 of treatment attributed to lymphoma.

## DISCUSSION

This study provides valuable insights into managing HBsAg-negative, anti–HBc-positive patients receiving rituximab-based chemotherapy. Although the study was underpowered due to incomplete enrollment, no clinically significant reactivation events occurred in either group. Somewhat surprisingly, both HBsAg reverse seroconversions occurred in the TAF arm, including one during ongoing antiviral therapy, raising concerns about medication adherence, especially given documented low-level HBV DNA weeks before HBsAg reappearance.

Previous studies have highlighted the risk of HBV reactivation in this patient population.^24^,^25^,^26^,^27^ Yeo et al.^28^ reported reactivation rates of up to 25%. Huang et al.^29^ randomized 80 patients to preemptive ETV or no therapy before rituximab-based chemotherapy for aggressive NHL, showing significantly lower rates of reactivation in the ETV group. Both studies used monthly HBV DNA monitoring, and patients randomized to placebo were started on antiviral therapy at the first appearance of HBV DNA. Using this approach, no adverse clinical outcomes were observed in either group. There were no episodes of HBV-associated hepatitis, liver-related complications, or interruptions to cancer therapy. Although both studies concluded that preemptive therapy prevents HBV reactivation, on-demand therapy was also effective in preventing clinically relevant reactivation. Because frequent HBV DNA testing is not typically available, our goal was to assess whether a simpler strategy of HBsAg and ALT monitoring during standard oncology follow-up would be equally effective.

Kusumoto et al.^30^ reported an overall HBV reactivation rate of 8.2%, and while lower with prophylactic antiviral therapy, events occurred despite prophylaxis (2.1% vs. 10.8%). Similar to our results, reactivation was limited to HBV DNA elevations without associated hepatitis or other consequences. Thus, although antiviral prophylaxis can reduce the risk of HBV reactivation, it does not fully eliminate the risk, particularly of low-level HBV DNA positivity.

Low-level HBV DNA was not uncommon, occurring in 50% of participants in each treatment group, although primarily before starting or after stopping TAF. The study findings apply primarily to patients who are HBV DNA-negative at baseline. Notably, HBV DNA positivity during follow-up was not associated with clinical hepatitis and did not lead to HBsAg reverse seroconversion. Even HBV DNA >1000 IU/mL was not indicative of clinically relevant HBV reactivation. Only 1 of the HBV DNA elevations >1,000 IU/mL occurred during TAF therapy, as the other episodes in the TAF group were before TAF initiation (baseline) or in phase 3, after TAF withdrawal. As in our study, low-level “blips” of HBV DNA have not been found to be associated with future hepatitis flares, antiviral resistance, progressive liver injury, or other consequences, but may indicate imperfect adherence.^31^ This raises an important consideration of whether HBV reactivation should be defined solely based on HBV DNA positivity, as has been reported in most studies.^28^,^29^,^30^ Alternatively, reactivation may be limited to HBsAg reverse seroconversion, or HBV-associated hepatitis, in which both HBV DNA positivity and ALT elevation must be present. Notably, the proposed strategy includes HBV DNA testing in real-time in the setting of ALT elevation, which would ensure that the rare possibility of a HBsAg mutant that may not be detected by HBsAg testing alone would not be missed. Our data support monthly HBsAg and ALT monitoring during chemotherapy and every 6–12 weeks post-chemotherapy as a safe strategy rather than more frequent HBV DNA testing, as both HBsAg reverse seroconversions were promptly treated without clinical consequence. However, given the small sample size, validating this approach in larger studies would be important before wide adoption.

Most guidelines recommend the use of ETV as first-line therapy to prevent HBV reactivation due to its safety profile and low risk of resistance compared with lamivudine.^18^,^32^,^33^ TDF is associated with a risk of renal toxicity and metabolic bone disease, both of which may be more frequent in patients undergoing cancer chemotherapy, many of whom are older with comorbidities. We found that TAF, which contains the same active antiviral compound but is associated with much less frequent renal or bone toxicity than TDF,^34^ was safe and well-tolerated in this population without any drug-related toxicity noted. The fact that we observed low-level HBV DNA positivity despite TAF suggests that adherence is an important consideration, as it is very improbable that these HBV DNA blips represented true viral breakthrough. Resistance testing was not possible because the HBV DNA levels were too low for sequencing. However, development of resistance is unlikely given the extremely high barrier to resistance of tenofovir.

Unfortunately, slow enrollment led to early study termination, limiting the power to assess the primary endpoint. Nonetheless, these data provide important insights into the safety of HBsAg monitoring as an alternative to HBV DNA testing. Our findings, combined with prior studies, support close monitoring with prompt antiviral initiation upon HBsAg reverse seroconversion as a viable alternative to prophylactic antiviral therapy in HBsAg-negative, anti-HBc-positive patients receiving anti-CD20 lymphoma-based chemotherapy. The observation that HBV DNA detectability and HBsAg reverse seroconversion occurred in TAF-treated patients highlights the need for careful monitoring with emphasis on adherence to both therapy and follow-up. The preferred duration of antiviral therapy and monitoring is unknown, but late HBV reactivations have been reported after rituximab-based therapy. A trial to evaluate the optimal duration of antiviral therapy to prevent reactivation will be very challenging to perform. Therefore, whenever antiviral therapy is discontinued, 6–12 months or even longer after stopping immunosuppression, close monitoring is required to detect and manage treatment withdrawal flares. Indeed, fatal reactivations have been reported after withdrawal of effective antiviral prophylaxis, and 1 of the 2 cases of HBsAg reverse seroconversion in this study occurred following TAF discontinuation.^35^ The optimal screening strategy after antiviral withdrawal remains undetermined. This study used testing every 6 weeks for 6 months post-therapy, followed by continued HBsAg testing at scheduled oncologic follow-up (every 3 months), which was effective in this small cohort.

Overall, this study showed that TAF is a safe antiviral for prophylaxis in HBsAg-negative, anti–HBc-positive patients receiving rituximab-based chemotherapy. HBsAg and ALT testing, rather than routine HBV DNA monitoring, effectively identified HBV reactivation in both patients who did and did not receive prophylaxis. Routine HBsAg and ALT monitoring with selective HBV DNA testing may be an alternative to universal prophylaxis in well-monitored settings. The study also highlights the importance of strict adherence to antiviral prophylaxis if it is used and close follow-up after stopping antiviral treatment due to the risk of withdrawal flares.

## Supplementary Material
