# Supplementary material for: Tenofovir alafenamide for prevention of HBV reactivation in HBsAg-negative, anti–HBc-positive patients undergoing rituximab-based chemotherapy: A multicenter randomized controlled trial
Source: Hepatol Commun. 2025 Dec 3;9(12):e0859. doi: 10.1097/HC9.0000000000000859 (PMC12674154; doi:10.1097/HC9.0000000000000859)
Supplement: Supplementary file 1 [file hc9-9-e0859-s001.docx]

**Supplemental Table 1. Baseline characteristics of patients with HBV DNA >1,000 IU/mL.**

| **Characteristic** | **Total** (n = 6) | **TAF** (n = 3) | **Placebo** (n = 3) |
| --- | --- | --- | --- |
| Age, years, median (IQR) | 59.2 (46.9 – 67.3) | 55.8 (35.8 – 67.3) | 62.6 (46.9 – 67.4) |
| Male, n (%) | 2 (33.3) | 1 (33.3) | 1 (33.3) |
| Race, n (%) |  |  |  |
| White | 2 (33.3) | 2 (66.7) | 0 (0) |
| Asian | 3 (50.0) | 1 (33.3) | 2 (66.7) |
| Lymphoma type, n (%) |  |  |  |
| Indolent | 4 (66.7) | 1 (33.3) | 3 (100) |
| Aggressive | 2 (33.3) | 2 (66.7) | 0 (0) |
| ALT, U/L, median (IQR) | 22.0 (12.0 – 40.0) | 15.0 (12.0 – 99.0) | 29.0 (8.0 – 40.0) |
| ALT, ULN, median (IQR) | 0.6 (0.3 – 1.0) | 0.4 (0.3 – 2.5) | 0.7 (0.2 – 1.0) |
| Direct bilirubin ^a^, µmol/L, median (IQR) | 2.0 (1.5 – 5.0) | 1.5 (1.0 – 2) | 5.0 (2.0 – 8.0) |
| Total bilirubin ^a^, µmol/L, median (IQR) | 6.0 (4.0 – 6.0) | 6.0 (6.0 – 6.0) | 4.0 (4.0 – 24.0) |
| Creatinine, µmol/L, median (IQR) | 65.0 (56.0 – 84.0) | 56.0 (32.0 – 84.0) | 66.0 (64.0 – 90.0) |
| anti-HBs positive, n (%) | 6 (100) | 3 (100) | 3 (100) |
| HBV DNA detectable, n (%) | 1 (16.7) ^b^ | 1 (33.3) ^b^ | 0 (0) |

^a^ Direct bilirubin was missing for 2 patients, and total bilirubin was missing for 1 patient.

^b^ Quantitative HBV DNA at baseline was 4,260 IU/mL for this patient.

ALT, Alanine aminotransferase; IQR, Interquartile range; SD, Standard deviation; TAF, Tenofovir alafenamide; ULN, Upper limit of normal.

**Supplemental Figure 1. Creatinine levels by arm.**

HBsAg, Hepatitis B surface antigen; HBV, Hepatitis B virus; TAF, Tenofovir alafenamide.

**Supplemental Figure 2. Quantitative HBV DNA levels by arm and lymphoma type.**

HBsAg, Hepatitis B surface antigen; HBV, Hepatitis B virus; TAF, Tenofovir alafenamide.

**Supplemental Figure 3. ALT levels by arm and lymphoma type.**

ALT, Alanine aminotransferase; HBsAg, Hepatitis B surface antigen; HBV, Hepatitis B virus; TAF, Tenofovir alafenamide.
